# Tumor invasion depth is a useful pathologic assessment for predicting outcomes in cervical squamous cell carcinoma after neoadjuvant radiotherapy

**DOI:** 10.1186/s13000-015-0426-6

**Published:** 2015-11-04

**Authors:** Yang Lv, Ning Wang, Yixiong Liu, Xia Li, Linni Fan, Mingyang Li, Lu Wang, Zhou Yu, Qingguo Yan, Ying Guo, Shuangping Guo, Lichun Wei, Mei Shi, Zhe Wang

**Affiliations:** State Key Laboratory of Cancer Biology, Department of Pathology Xijing Hospital, Fourth Military Medical University, Xi’an, Shaanxi Province 710032, P.R. China; Department of Radiation Oncology, Xijing Hospital, Fourth Military Medical University, Xi’an, Shaanxi Province 710032, P.R. China

**Keywords:** Pathologic assessment, Cervical squamous cell carcinoma, Neoadjuvant radiotherapy, Chemoradiotherapy, Tumor invasion depth, Cervical internal surface

## Abstract

**Background:**

To evaluate whether tumor invasion depth can be a reliable and easily applicable pathologic assessment strategy to predict outcomes using surgically resected cervical squamous cell carcinoma specimens from patients who have received neoadjuvant radiotherapy (RT) or concurrent chemoradiotherapy (CCRT).

**Methods:**

We included 173 patients with cervical squamous cell carcinoma who received neoadjuvant CCRT (*n* = 125) or RT (*n* = 48) and underwent subsequent radical hysterectomy. Data for the pre-operative clinical International Federation of Gynecology and Obstetrics (FIGO) stage, post-operative pathologic FIGO stage, World Health Organization (WHO) double diameter measurement evaluation, response evaluation criteria in solid tumors (RECIST 1.1) criteria, tumor necrosis rate (TNR), and tumor regression grade (TRG) were investigated to identify correlations with outcomes related to distant metastasis and survival. The tumor invasion depth (TID) and the tumor invasion depth with cytokeratin immunostaining correction (TIDC) at the cervical internal surface were measured to assess their relations to patients’ outcomes.

**Results:**

Based on measurements taken via transvaginal ultrasound, the pre-operative clinical and post-operative pathologic FIGO staging as well as the WHO double diameter measurement evaluation and RECIST 1.1 criteria were predictive of distant metastasis and survival-related outcomes. Also, lymph node involvement was found to be an independent prognostic factor for recurrence and distant metastasis. Finally, univariate analysis showed both the TID and TIDC were highly related to distant metastasis, overall survival, and progression-free survival, irrespective of the clinical stage of carcinomas.

**Conclusion:**

The TID or TIDC measured at the cervical internal surface is a useful and easily applied pathologic prognostic factor for distant metastasis and survival outcomes in patients with cervical squamous cell carcinoma treated with neoadjuvant RT or CCRT.

**Electronic supplementary material:**

The online version of this article (doi:10.1186/s13000-015-0426-6) contains supplementary material, which is available to authorized users.

## Background

Cervical squamous cell carcinoma is one of the most common gynecological malignancies, affecting the health of women worldwide [1, 2]. In China, cervical carcinoma is the second most common gynecological malignancy [3]. The treatment strategy for cervical carcinoma has been changed significantly in the past two decades [4, 5]. For International Federation of Gynecology and Obstetrics (FIGO) stage Ib and II patients, treatment involving neoadjuvant therapies plus surgery have been shown to achieve more benefits than surgery alone [6–8]. In addition, just as we have observed in our clinical practice, previous studies also have confirmed that even FIGO stage III b cervical carcinoma patients who receive neoadjuvant therapies are able to endure surgical treatment with better outcomes [9–11]. Moreover, the use of neoadjuvant radiotherapy (RT) alone or with concomitant chemoradiotherapy (CCRT) is effective for achieving excellent cervical carcinoma control along with surgical resection [12, 13].

Pathologic findings in resected specimens of cervical carcinoma can be the basis for extremely accurate assessment of the extent of disease [14, 15]. However, as neoadjuvant RT and CCRT are applied in increasingly more cervical carcinoma patients, the need for a useful and easily applied pathologic assessment becomes increasingly critical.

In the present study, we conducted a retrospective analysis of 173 cervical squamous cell carcinoma patients who received neoadjuvant RT or CCRT with the objective of determining whether the tumor invasion depth could be an objectively, reproducibly, and quantitatively measured pathologic parameter to predict these patients’ outcomes.

## Methods

### Patients

Between April 2006 and June 2011, 173 FIGO stage Ib2–IIIb cervical carcinoma patients underwent radical hysterectomy and pelvic lymph node dissection after neoadjuvant RT (*n* = 48) or CCRT (*n* = 125) in the Xijing Hosptial, Fourth Military Medical University. The interval between pre-operative RT or CCRT and radical surgery was typically 2–3 weeks. The pathologic subtype in all cases was squamous cell carcinoma. Patients’ complete information was recorded in our department, and follow-up information was obtained from electronic medical records or by telephone interviews of patients between April 2006 and December 2014.

And all the researches, including the different treatments, the name of the patients and their outcomes, were performed with the approval of an appropriate ethics committee of Fourth Military Medical University.

### Cervical carcinoma staging

We evaluated target lesions via physical examination and transvaginal ultrasound (TVS) examination, and tumor size was measured according to images taken before surgery. Based on the 2009 FIGO staging system [16, 17], the pre-operative clinical staging data included primarily the size of the imaged tumor and observations from physical examinations.

After surgery, tumor size and extent were evaluated by pathologic examination. Post-operative staging was carried out as “y” Pathologic stage according to 2009 FIGO staging system.

### Tumor neoadjuvant treatment response evaluation

The tumor size was measured by TVS examination before RT and again before surgery, and the data were used for evaluation by World Health Organization (WHO) double diameter measurement [18] and the response evaluation criteria in solid tumors (RECIST) 1.1 criteria [19]. The microscopic residual tumor size was used to evaluate the tumor necrosis rate (TNR) [20] and tumor regression grade (TRG) evaluation.

Considering the change in tumor burden as an important feature in the clinical evaluation of cancer therapeutics, the WHO double diameter measurement was obtained as previously described [18]. Complete response (CR) is defined as the disappearance of all target lesions, and any pathological lymph nodes must be reduced to having a short axis <10 mm. Partial response (PR) is defined as at least a 50 % decrease in the products of bidimensional lesion measurements of target lesions. Progressive disease (PD) is defined as at least a 25 % increase and stable disease (SD) is defined as tumor shrinkage not sufficient to qualify as PR or tumor growth not sufficient to qualify as PD.

The response evaluation criteria in solid tumors (RECIST) 1.1 criteria [19] use a unidimensional measure (the longest diameter) assessment of one or more target lesions with summation of unidimensional assessments of many lesions to quantify measurable tumor lesions. CR requires a complete absence of disease, whereas PR indicates a more than 30 % decrease in the sum of the largest diameters. PD is defined by an increase in the sum of the largest diameters by more than 20 %, and SD represents all the outcomes between PR and PD.

The TNR [20] is defined as the presence of microscopic coagulative necrosis, and the presence of necrosis on gross examination was not considered. The extent of tumor necrosis was assessed in a semiquantitative manner at low magnification (magnification, ×40) of all available tumor blocks. The TRG was recorded according to a 5-grade scale: TRG 1 indicates absence of residual cancer; TRG 2 indicates few residual cancer cells; TRG 3 indicates a considerable number of residual cancer cells; TRG 4 indicates outgrowth of residual cancer; and TRG 5 indicates a lack of regressive changes.

### Neoadjuvant RT and chemotherapy regimens

Pre-operative pelvic RT was delivered using three-dimensional conformal radiation techniques and 6.0 or 15.0 medium voltage photons using a linear accelerator (Clinac 23EX or 600 C/D; Varian Medical Systems, Palo Alto, CA, USA). Patients were immobilized in the supine position using a custom vacuum mattress and first underwent a computed tomography (CT) simulation scan (PQS + AcQSim; Philips, Amsterdam, The Netherlands) with intravenous contrast, using 5.0-mm slice thickness. Simulation images extended from L1 to 5.0 cm below the ischial tuberosities. The treatment planning was designed and computed using the Plato system version 2.7.5 (Varian, Salt Lake City, UT, USA). The pelvic irradiation dose was 40–50 Gy in 20–25 fractions.

CCRT was administered to 121 patients via weekly intravenous infusion of cisplatin (40 mg/m^2^) during pelvic external beam RT. Patients received either three (*n* = 30, 25 %), four (*n* = 46, 38 %), or five (*n* = 45, 37 %) cycles of cisplatin. Chemotherapy was withheld under the following conditions: white blood cell count <2.0 × 10^9^/L, absolute neutrophil count <1.0 × 10^9^/L, platelet count <50 × 10^9^/L, or grade 3–4 radiation enteritis or cystitis.

The interval between pre-operative RT or CCRT and radical surgery was 3 weeks for 71 (41.0 %) patients, 2 weeks for 93 (53.8 %) patients, and 1 week for only 9 (5.2 %) patients.

### Histopathologic examination and immumohistochemical staining

The histopathologic response to neoadjuvant therapy was evaluated based on pathologic examination of resected specimens, including uterine tissue, the vaginal cuff, the parametrium, bilateral adnexa, and pelvic lymph nodes. The status of resected lymph nodes was described in terms of lymph-node involvement (involved or not involved).

The pan-cytokeratin antibody (AE1/AE3) (Dako, Glostrup, Denmark) was used in immunohistochemical staining to identify any invisible residual tumor cells.

### Measurement of TID

**Fig. 1:**
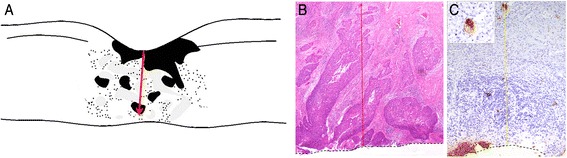
**a** Sketch illustrating the measurement method of tumor invasion depth (TID) at the cervical internal surface. **b** The red arrow indicates the measurement of the TID at the cervical internal surface in a representative specimen (magnification, ×40). **c** The yellow arrow indicates the measurement of TIDC at the cervical internal surface in a representative specimen with cytokeratin immunostaining correction (magnification, ×40)

Pathologic examination of specimens removed by radical hysterectomy was performed using a standardized process. Briefly, each fresh surgical specimen was opened with a scissor at the 12:00 o'clock position and pinned on a cork plate with fixation overnight in 4 % buffered formalin. Then the TID (in millimeters) was measured on the H&E stained slides with the largest tumor area by two pathologists blinded to the outcomes of the patients using magnifying ruler. The TID was recognized as the longest tumor length perpendicular to the cervical internal surface (Fig. 1a & 1b). With the cytokeratin immunostaining correction, the TIDC also was measured as the length perpendicular to the cervical internal surface to the farmost residual tumor cells identified by AE1/AE3 immunostaining (Fig. 1c).

### Statistical analysis

Quantitative variables are presented as frequencies and percentages, and the Chi-square test and Spearman test were applied to assess associations between categorical variables. Univariate analysis was first performed to identify factors of strongest significance, followed by step-wise stratified analysis and complemented with multivariate analysis to define the best statistically significant combination of these factors. Then multivariate Cox proportional hazard models were applied to assess the independent impacts of combined variables with distant metastasis, progression-free survival (PFS), and overall survival (OS), followed by step-wise stratified analysis beginning with the strongest factors.

Receiver operating characteristic (ROC) curves were calculated to represent the relationship between true positive events (sensitivity) and false positive events (100 – specificity) for different cut-off points. Such ROC curves for sensitivity and specificity are often used to assess the performance of diagnostic tests, prognostic factors, and classifiers.

Therefore, ROC curves were constructed for quantitative variables to determine the best cut-off point values discriminating between groups with good or poor prognosis and to define the distinctions between groups with differing prognosis.

OS and PFS were calculated from the date of diagnosis to the date of the last follow-up. Death events in the absence of disease progression were censored in the calculation of PFS. Survival probabilities were calculated by the Kaplan–Meier method and compared by the log-rank test. A *P*-value of less than 0.05 was considered statistically significant in all tests, and all statistical analyses were performed using SPSS version 19.0 software (IBM, Armonk, NY, USA).

## Results

### Patient characteristics

The median age of all 173 patients was 46 years (range, 25–71 years), and the majority of patients (150/173) had FIGO stage IIb cervical carcinoma. Detailed data regarding patient characteristics before and after radical hysterectomy are presented in Table 1. Notably, the incidence rates of death within 5 years of diagnosis (6/15, 40 %; *P* < 0.001) and the development of distant metastasis (5/15, 33.3 %; *P* < 0.05) were significantly greater in patients with stage IIIb cervical carcinoma than in patients with carcinomas of other stages. The results also showed that distant metastasis and death occurred more frequently among patients who experienced PR compared to CR, (*P* < 0.05 for WHO double diameter measurement and the RECIST 1.1 evaluation). Finally, lymph node metastasis also was significantly associated with distant metastasis and recurrence (*P* < 0.05).

**Table 1:** The characteristics of 173 patients with FIGO stages Ib-IIIb cervical squamous cell carcinoma before and after the radical hysterectomy and results of correlation analyses

Characteristic		Number of patients, n (%)	Median age, years (range)	Distant metastasis, n (%)	*P*	Recurrence, n (%)	*P*	The death events within 5 years, n (%)	*P*
Treatments	CCRT	125(72.3 %)	45(25–65)	12(9.6 %)	0.579	4(3.2 %)	0.697	7(5.6 %)	0.512
	RT	48(27.7 %)	46(34–71)	6(12.5 %)		1(2.1 %)		4(8.3 %)	
FIGO stage	Ib	5(2.9 %)	45(42–47)	0	0.003	0	0.319	0	<0.001
	IIa	3(1.7 %)	45(38–58)	0		0		0	
	IIb	150(86.7 %)	45(27–71)	13(8.7 %)		4(2.7 %)		5(3.3 %)	
	IIIb	15(8.7 %)	48(25–65)	5(33.3 %)		1(6.7 %)		6(40 %)	
yPathologic stage,	*	84(48.6 %)	45(27–65)	3(3.6 %)	0.001	1(1.2 %)	0.150	1(1.2 %)	0.003
	yI a1	24(13.9 %)	42(35–52)	2(8.3 %)		0		1(4.2 %)	
	yI a2	5(2.9 %)	43(32–57)	0		1(20 %)		1(20 %)	
	yI b2	61(35.2 %)	46(25–71)	13(21.3 %)		3(4.9 %)		8(13.1 %)	
WHO evaluation	CR	83(48.0 %)	45(27–65)	3(3.6 %)	0.007	1(1.2 %)	0.206	1(1.2 %)	0.010
	PR	88(50.9 %)	45(25–65)	15(17.0 %)		4(4.5 %)		10(11.4 %)	
	SD	2(1.2 %)	57(43–71)	0		0		0	
	PD	0	0	0		0		0	
RECIST1.1	CR	83(48.0 %)	45(27–65)	3(3.6 %)	0.005	1(1.2 %)	0.224	1(1.2 %)	0.007
	PR	90(52.0 %)	45(25–71)	15(16.7 %)		4(4.4 %)		10(11.1 %)	
	SD	0	0	0		0		0	
	PD	0	0	0		0		0	
TNR	<90 %	102(59.0 %)	46(27–71)	11(10.8 %)	0.506	3(2.9 %)	0.789	8(7.8 %)	0.309
	>90 %	71(41.0 %)	45(25–65)	7(9.9 %)		2(2.8 %)		3(4.2 %)	
TRG	TRG1	23(13.3 %)	46(36–58)	1(4.3 %)	0.176	0	0.925	0	0.119
	TRG2	70(40.5 %)	45(27–71)	7(10 %)		3(4.3 %)		5(7.1 %)	
	TRG3	44(25.4 %)	44(25–65)	4(9.1 %)		1(2.3 %)		1(2.3 %)	
	TRG4	33(19.1 %)	47(30–65)	5(15.2 %)		1(3.0 %)		4(12.1 %)	
	TRG5	3(1.7 %)	45(39–45)	1(33.3 %)		0		1(33.3 %)	
Lymph node metastasis	yes	20(11.6 %)	46(37–65)	7(35.0 %)	0.000	3(15.0 %)	0.001	3(15.0 %)	0.093
	no	153(88.4 %)	45(25–71)	11(7.2 %)		2(1.3 %)		8(5.2 %)	

Note: Post-operative pathologic FIGO stage *: FIGO staging no longer includes Stage 0 (Tis)

### Outcomes according to existing tumor staging systems and evaluation standards

**Fig. 2:**
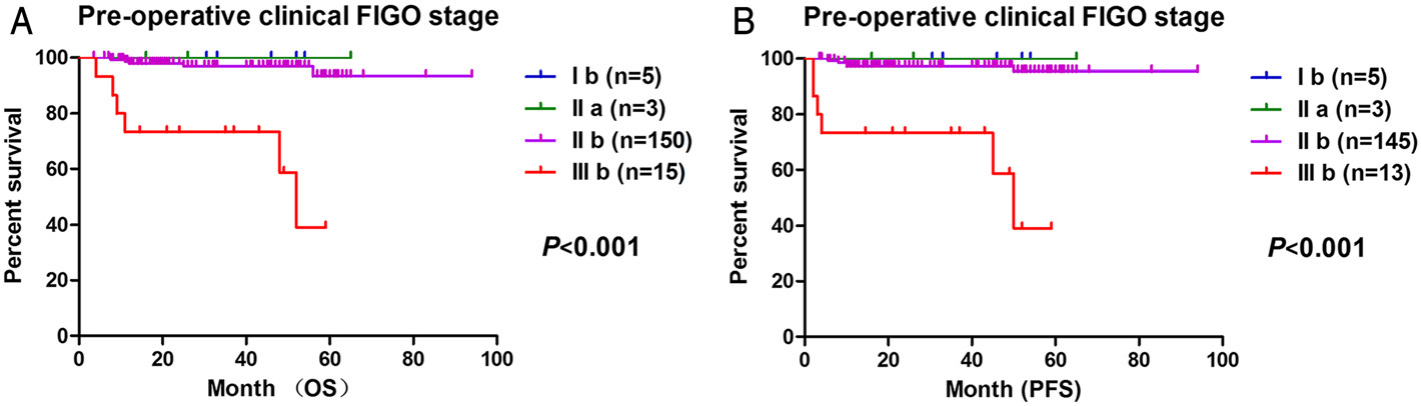
Association of overall survival (OS) and progression-free survival (PFS) with the pre-operative clinical FIGO stage. **a** OS and **b** PFS curves show a slight difference with different pre-operative clinical FIGO staging prior to radical hysterectomy

Analyses of the results in our study indicate that both the pre-operative clinical FIGO stage (Fig. 2, Table 1) and post-operative pathological FIGO stage can predict survival-related outcomes (Table 1, Additional file 1: Figure S1). In addition, the existing tumor evaluation standards, specifically the WHO double diameter measurement evaluation and the RECIST 1.1, were also correlated with the outcomes of our patients (Additional file 2: Figures S2 & Additional file 3: Figure S3 and Table 1). However, the TNR and TRG failed to be prognostic indicators according to Spearman tests (Table 1).

### Outcomes according to TID and TIDC

Along with the factors identified in univariate analysis as potentially influencing patient outcomes, we included the TID (*P* < 0.05, relative hazard [RH] = 9.7, 95 % confidence interval [CI] = 1.508–32.350) in the multivariate Cox proportional hazard models as an independent prognostic factor due to a strong correlation with distant metastasis, PFS, and OS. The results indicate that TID from the cervical internal surface represents a novel predictor of prognosis in cervical carcinoma. Based on the statistical differences in distant metastasis and survival outcomes according to TID, a ROC curve was generated to investigate the best cut-off value for classifying TID. From this curve, the best cut-off value was found to be 3.75 mm. The same calculations for TIDC resulted in an adjusted cut-off value of 4.75 mm. The results of correlation analyses of patient data according to these cut-off values are presented in Table 2.

**Table 2:** Patient characteristics according to TID and TIDC cut-off values and results of correlation analyses

Characteristic	Invasion depth (mm)	Number of patients, n (%)	Median age, years (range)	Distant metastasis, n (%)	*P*	Lymph node metastasis, n (%)	*P*	Recurrence (%)	*P*	The death events in 5 years, n (%)	*P*
TID	<3.75	104 (60.1 %)	45 (27–71)	4 (3.8 %)	<0.001	12 (11.5 %)	0.991	1 (1.0 %)	0.064	1 (1.0 %)	<0.001
	>3.75	69 (39.9 %)	46 (25–65)	14 (20.2 %)		8 (11.6 %)		4 (5.8 %)		10 (14.5 %)	
TIDC	<4.75	100 (57.8 %)	45 (27–65)	4 (4.0 %)	0.001	12 (12.0 %)	0.834	1 (1.0 %)	0.083	1 (1.0 %)	0.001
	>4.75	73 (42.2 %)	46 (25–71)	14 (19.2 %)		8 (11.0 %)		4 (5.5 %)		10 (13.7 %)	

TID or TIDC values less than the cut-off values could represent two different situations: (1) a complete lack of residual tumor cells, and (2) a measurable invasion depth less than the cut-off point. Correlation analysis indicated that there were no statistical differences in outcomes related to survival (*P* = 0.254) or distant metastasis (*P* = 0.212) between these situations. The same results were obtained for TIDC, with no differences in outcomes related to survival (*P* = 0.823) and distant metastasis (*P* = 0.412).

**Fig. 3:**
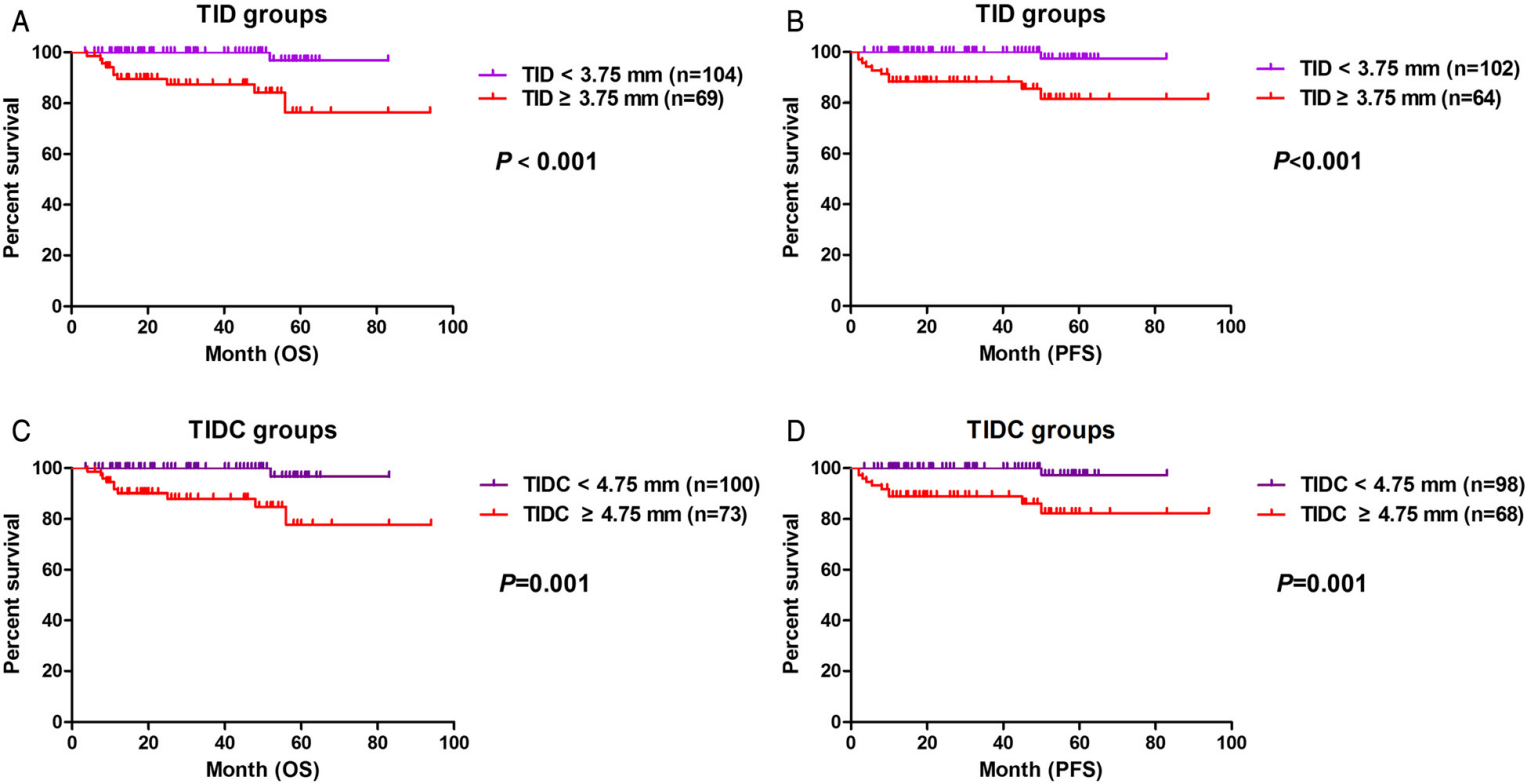
Association of overall survival (OS) and progression-free survival (PFS) with the TID and TIDC at the cervical internal surface. **a** OS and **b** PFS curves show a clear difference between patient outcomes according to a TID cut-off value of 3.75 mm (TID <3.75 mm and TID ≥3.75 mm). **c** OS and **d** PFS curves show the prognostic significance of TIDC with the cut-off point of 4.75 mm (TIDC <4.75 mm and TIDC ≥4.75 mm)

The prognostic value of the TID and TIDC cut-off values for distant metastasis, PFS, and OS was confirmed by Kaplan–Meier survival curves (Fig. 3). Thus, a TID greater than 3.75 mm and a TIDC greater than 4.75 mm on immunostained specimens indicate a higher relative risk of distant metastasis and poor survival.

**Fig. 4:**
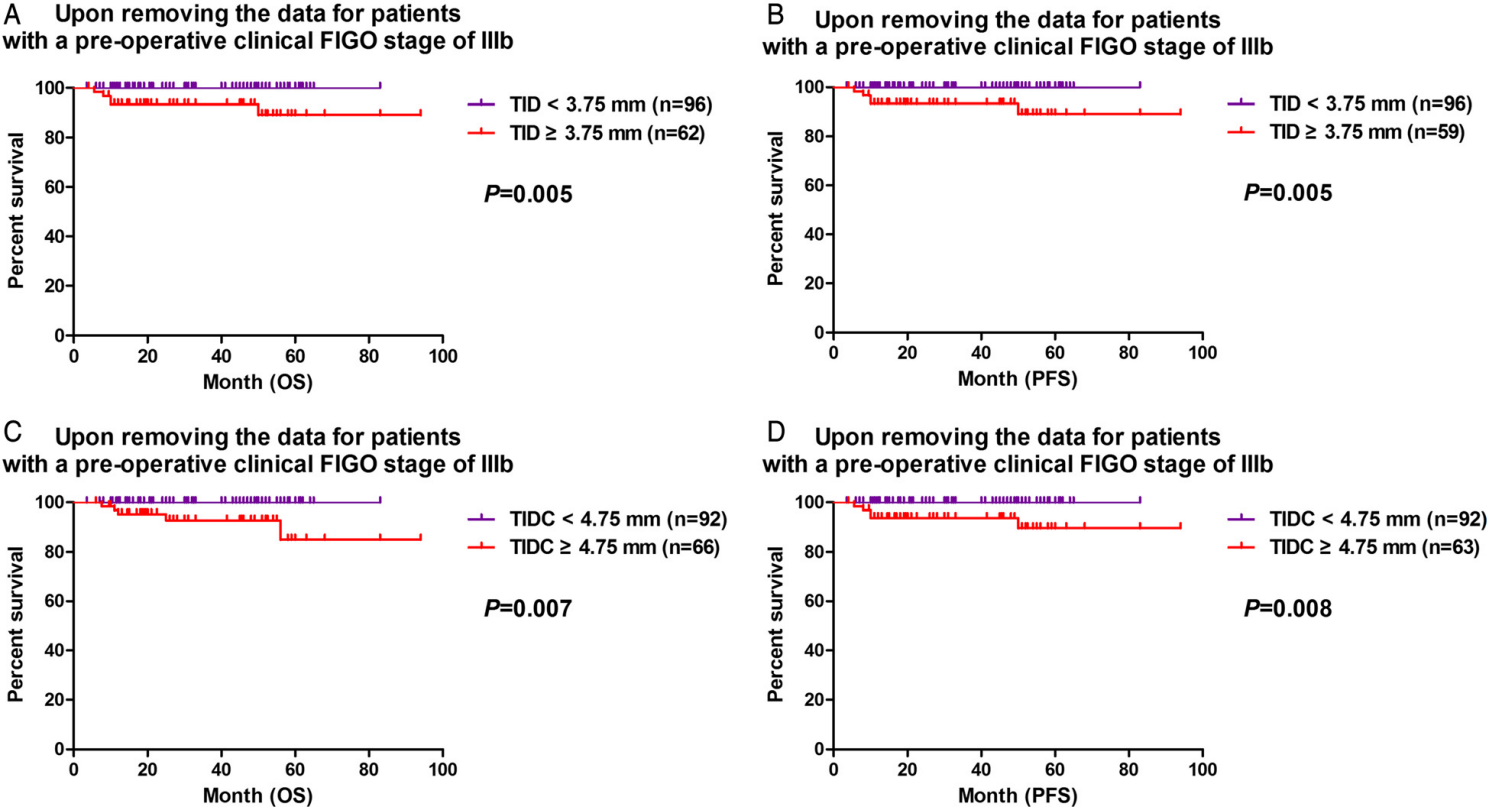
Upon removing the data for patients with a pre-operative clinical FIGO stage of IIIb, **a** OS and **b** PFS curves continue to show the significant prognostic value of TID with a cut-off value of 3.75 mm. **c** OS and **d** PFS curves generated while excluding data for patients with stage IIIb carcinoma also continue to demonstrate the prognostic significance of the TIDC with a cut-off value of 4.75 mm

Because the results shown in Fig. 2 demonstrated that patients with pre-operative clinical FIGO stage IIIb cancer had a lower 5-year survival rate than patients with other stages and the Kaplan–Meier survival curves could not show clear statistical differences between the survival outcomes of stage Ib and II patients, we further analyzed the predictive value of the TID and TIDC cut-off values excluding data for patients without the clinical stage IIIb carcinoma to eliminate the effects of the clinical stage of cervical carcinoma. The results showed that these TID and TIDC cut-off values were still prognostic indicators for PFS and OS (Fig. 4).

## Discussion

In this study, we assessed the prognostic value of different clinical and pathologic assessment parameters using data of 173 cervical squamous cell carcinoma patients who underwent neoadjuvant RT or CCRT prior to radical hysterectomy. Although no standard procedures for neoadjuvant therapy plus surgery have been confirmed yet and more supporting data are still needed, especially data regarding CCRT, some studies have suggested that pre-operative CCRT is associated with significantly improved PFS and OS compared with RT alone [21, 22], whereas other studies have provided evidence suggesting almost the same clinical outcomes following these different treatment modalities [13, 23]. Our data also did not show significant differences in the outcomes of patients who received RT alone or CCRT, which is consistent with the results of the study by Keys et al. [24]. Moreover, improvement of the 5-year OS rate with neoadjuvant CCRT over that achieved with RT alone remains to be proven. In our study, the 60.0 % 5-year survival rate of stage IIIb patients is similar to the rates previously reported, whereas the 96.7 % 5-year survival rate of stage IIb patients in our study seems to be higher than rates previously reported (e.g., 71.0 %) [25, 26]. These findings may suggest the benefit of neoadjuvant RT or CCRT, but further studies are still needed to draw such conclusions.

Before radical hysterectomy, we obtained the tumor size measurements used in the clinical FIGO staging system and WHO and RECIST 1.1 evaluation criteria by TVS examination, which has been shown to be highly accurate for detecting tumor presence and evaluating the local extension of cervical carcinoma [27, 28]. Because this imaging technique is faster, cheaper, and more widely available than other imaging techniques [27, 28], its use is consistent with our objective of establishing prognostic criteria for cervical carcinoma underwent neoadjuvant treatment that are easily applied in most hospitals. However, TVS is a painful and time-consuming procedure, especially for the patients who have undergone RT or CCRT.

The FIGO staging system [16, 17], especially the revised FIGO (2009) staging system, is widely used in standard clinical and pathologic staging procedures for many types of cancer, including cervical carcinoma. Similar to the results of previous clinicopathologic studies on cervical carcinoma [29], our data demonstrate that the clinical FIGO staging prior to radical hysterectomy serves as a valuable independent predictor of distant metastasis and survival outcomes (Table 1 and Fig. 2) in patients with FIGO stage III carcinomas, whereas it is not a very effective predictor of outcomes for stage I and II carcinomas. In addition, our results showed that post-operative pathologic FIGO staging is a less effective predictor compared with the clinical stage. Use of the FIGO staging system both before and after surgical treatment are dependent on the assessment of tumor size and invasion of the parametrium and pelvic side wall as well as the evaluation of adjacent organs and lymph node involvement [30]. Thus, high accuracy of FIGO staging prior to radical hysterectomy for cervical carcinoma prognosis is not easily obtainable and depends heavily on accurate clinical examinations [28–32].

The WHO (double diameter measurement evaluation) criteria and RECIST 1.1 criteria are widely used and also were found to be effective in our study (Table 1 and Additional file 2: Figure S2 & Additional file 3: Figure S3). However, these two sets of criteria clearly state that tumor lesions situated in a previously irradiated area, or in an area subjected to other loco-regional therapy, are not usually considered measurable unless evidence of progression in the lesion has been demonstrated [31]. Moreover, both sets of criteria also suggest the ultrasound examinations should not be used for imaging-based evaluation measurements, because ultrasound is regarded as operator dependent and therefore of limited reliability [32]. However, the effective application of these two sets of criteria in our study indicated that the use of TVS is more reliable than ordinary ultrasound examination with respect to the accuracy of the results.

In our study, to find useful prognostic factors for cervical carcinoma treated with neoadjuvant RT or CCRT, we also included the TNR in our analyses and showed that the TNR cannot be considered an effective prognostic factor (Table 1). This is likely because the degree of necrosis within tumor tissue is not apparent until some time after treatment. Moreover, TNR also can be easily affected by tumor heterogeneity, artery dilatation, and vascular proliferation within the tumor [33]. We also applied the 5-point TRG scoring system to determine whether the TRG is correlated with outcomes in these patients. Based on the hypothesis that a shorter tumor cell doubling time indicates a more aggressive tumor, the regression grade is in general considered to be related to patients’ prognosis. However, the TNR is a subjective criterion not based on an objective measurement and thus does not offer good reproducibility, and our results showed no clear prognostic significance for patients with cervical carcinoma treated with neoadjuvant therapy (Table 1).

Residual tumor cells are generally considered aggressive, and the area containing such cells may be important for the determination of invasive and metastatic capacity. Dipen et al. proposed “tumor thickness” as a new prognostic factor for therapy response and survival outcome in patients with resected hepatic colorectal metastases [34], and other studies also indicated that the tumor thickness remains the most important determinant of melanoma patient survival [35, 36]. For cervical carcinoma, tumor diameter and volume, as determined by pre-surgery imaging examinations, can predict progression-free survival [37], and previous studies confirmed that the tumor size on T2-weighted magnetic resonance images is a useful index for evaluating parametrial involvement of cervical carcinoma with full-thickness stromal invasion [38]. Therefore, based on the hypothesis that the existence of residual tumor cells correlates with the outcomes of patients, the TID was designed to measure the length perpendicular to the cervical internal surface in the maximal cutting plane. Measurement of this dimension was less time consuming and more reproducible, because few parameters are considered and major parts of the tumor cannot be disregarded [34, 39].

In practice, nest-like or cord-like scattered tumor cells and continuous tumor tissue invading to different depths with different degrees of necrosis and inflammatory reactions can be identified easily under a microscope. Also, the reproducibility of such objective measurements could not be easily influenced by the pathologist’s experience. In the present study, cytokeratin immunostaining was used to identify some uncertain residual tumor tissue and obtain TIDC measurements. The positive results demonstrated the existence of residual tumor tissue and inevitably changed the measured invasion depth. Even after we eliminated the major influence of patients’ pre-operative clinical FIGO stages on survival outcomes, both the TID and TIDC were found to be significantly correlated with distant metastasis and survival outcomes in cervical squamous cell carcinoma patients who had undergone adjuvant RT or CCRT.

## Conclusion

Pre-operative clinical FIGO staging as well as the WHO double diameter measurement evaluation and RECIST 1.1 criteria, according to tumor size assessed by TVS examination, were found to be useful predictive indicators of patient outcomes following radical hysterectomy in cervical squamous carcinoma patients who underwent neoadjuvant RT or CCRT. However, in addition to these previously reported assessment standards and methods, our results also provide evidence that the TID or TIDC at the cervical internal surface is a useful and easily applied prognostic factor for distant metastasis and survival outcomes in these patients, irrespective of pre-operative FIGO staging.

## Additional files

Additional file 1: Figure S1. Association of overall survival (OS) and progression-free survival (PFS) with the post-operative “y” Pathologic stage. (A) OS and (B) PFS curves show a slight difference with different post-operative “y” Pathologic staging after the radical hysterectomy. Note: Post-operative pathologic FIGO stage *: FIGO staging no longer includes Stage 0 (Tis). (ZIP 544 kb) Additional file 2: Figure S2. Association of overall survival (OS) (A) and progression-free survival (PFS) (B) curves show a clear difference between patient outcomes according to the WHO double diameters measurement evaluation. Note: CR: Complete response, PR: Partial response, PD: Progressive disease, SD: Stable disease. (ZIP 423 kb) Additional file 3: Figure S3. Association of overall survival (OS) (A) and progression-free survival (PFS) (B) curves show a clear difference between patient outcomes according to response evaluation criteria in solid tumors (RECIST1.1). Note: CR: Complete response, PR: Partial response, PD: Progressive disease, SD: Stable disease. (ZIP 399 kb)

## References

[1] Intaraphet S, Kasatpibal N, Siriaunkgul S, Sogaard M, Patumanond J, Khunamornpong S, Chandacham A, Suprasert P (2013). Prognostic impact of histology in patients with cervical squamous cell carcinoma, adenocarcinoma and small cell neuroendocrine carcinoma. Asian Pac J Cancer Prev.

[2] Wang SS, Sherman ME, Hildesheim A, Lacey JV, Devesa S (2004). Cervical adenocarcinoma and squamous cell carcinoma incidence trends among white women and black women in the United States for 1976–2000. Cancer.

[3] Chen W, Zheng R, Zhang S, Zhao P, Zeng H, Zou X, He J (2014). Annual report on status of cancer in China, 2010. Chin J Cancer Res.

[4] Katanyoo K, Sanguanrungsirikul S, Manusirivithaya S (2012). Comparison of treatment outcomes between squamous cell carcinoma and adenocarcinoma in locally advanced cervical cancer. Gynecol Oncol.

[5] Demirci S, Ozsaran Z, Ozsaran A, Yavas F, Demircioglu B, Hanhan M, Dikmen Y, Aras AB (2012). Evaluation of treatment results and prognostic factors in early-stage cervical carcinoma patients treated with postoperative radiotherapy or radiochemotherapy. Eur J Gynaecol Oncol.

[6] Mikami M, Aoki Y, Sakamoto M, Shimada M, Takeshima N, Fujiwara H, Matsumoto T, Kita T, Takizawa K (2014). Surgical principles for managing stage IB2, IIA2, and IIB uterine cervical cancer (Bulky Tumors) in Japan: a survey of the Japanese Gynecologic Oncology Group. Int J Gynecol Cancer.

[7] Rydzewska L, Tierney J, Vale CL, Symonds PR (2012). Neoadjuvant chemotherapy plus surgery versus surgery for cervical cancer. Cochrane Database Syst Rev.

[8] Benedetti-Panici P, Greggi S, Colombo A, Amoroso M, Smaniotto D, Giannarelli D, Amunni G, Raspagliesi F, Zola P, Mangioni C, Landoni F (2002). Neoadjuvant chemotherapy and radical surgery versus exclusive radiotherapy in locally advanced squamous cell cervical cancer: results from the Italian multicenter randomized study. J Clin Oncol.

[9] Candelaria M, Cetina L, Perez-Cardenas E, de la Cruz-Hernandez E, Gonzalez-Fierro A, Trejo-Becerril C, Taja-Chayeb L, Chanona J, Arias D, Duenas-Gonzalez A (2010). Epigenetic therapy and cisplatin chemoradiation in FIGO stage IIIB cervical cancer. Eur J Gynaecol Oncol.

[10] Zuliani AC, Cunha Mde O, Esteves SC, Teixeira JC (2010). Brachytherapy for stage IIIB squamous cell carcinoma of the uterine cervix: survival and toxicity. Rev Assoc Med Bras.

[11] Wei LC, Wang N, Shi M, Liu JY, Li JP, Zhang Y, Huang YH, Li X, Chen Y (2013). Clinical outcome observation of preoperative concurrent chemoradiotherapy/radiotherapy alone in 174 Chinese patients with local advanced cervical carcinoma. Onco Targets Ther.

[12] Lee MY, Wu HG, Kim K, Ha SW, Kim JS, Kim IA, Lee HP (2007). Concurrent radiotherapy with paclitaxel/carboplatin chemotherapy as a definitive treatment for squamous cell carcinoma of the uterine cervix. Gynecol Oncol.

[13] Rydzewska L, Tierney J, Vale CL and Symonds PR. Neoadjuvant chemotherapy plus surgery versus surgery for cervical cancer. Cochrane Database Syst Rev 2010; CD007406. 10.1002/14651858.CD007406.pub2 20091632

[14] Zaino RJ (2000). Glandular lesions of the uterine cervix. Mod Pathol.

[15] Coucke PA, Maingon P, Ciernik IF, Phuoc DH (2000). A survey on staging and treatment in uterine cervical carcinoma in the Radiotherapy Cooperative Group of the European Organization for Research and Treatment of Cancer. Radiother Oncol.

[16] Serour GI (2010). A vision for FIGO 2009–2012. Int J Gynaecol Obstet.

[17] Yoon A, Park JY, Lee YY, Kim TJ, Choi CH, Bae DS, Kim BG, Lee JW, Nam JH (2014). Prognostic factors and outcomes in endometrial stromal sarcoma with the 2009 FIGO staging system: a multicenter review of 114 cases. Gynecol Oncol.

[18] Khokher S, Qureshi MU, Chaudhry NA (2012). Comparison of WHO and RECIST criteria for evaluation of clinical response to chemotherapy in patients with advanced breast cancer. Asian Pac J Cancer Prev.

[19] Watanabe H, Okada M, Kaji Y, Satouchi M, Sato Y, Yamabe Y, Onaya H, Endo M, Sone M, Arai Y (2009). New response evaluation criteria in solid tumours-revised RECIST guideline (version 1.1). Gan To Kagaku Ryoho.

[20] Kim MS, Lee SY, Cho WH, Song WS, Koh JS, Lee JA, Yoo JY, Jeon DG (2008). Tumor necrosis rate adjusted by tumor volume change is a better predictor of survival of localized osteosarcoma patients. Ann Surg Oncol.

[21] Houvenaeghel G, Lelievre L, Buttarelli M, Jacquemier J, Carcopino X, Viens P, Gonzague-Casabianca L (2007). Contribution of surgery in patients with bulky residual disease after chemoradiation for advanced cervical carcinoma. Eur J Surg Oncol.

[22] El-Weshi A, Khafaga Y, Allam A, Mosseri V, Ibrahim E, El-Serafi M, El-Badawi S (2001). Neoadjuvant chemotherapy plus conventional radiotherapy or accelerated hyperfractionation in stage III and IV nasopharyngeal carcinoma--a phase II study. Acta Oncol.

[23] Duenas-Gonzalez A, Lopez-Graniel C, Gonzalez-Enciso A, Mohar A, Rivera L, Mota A, Guadarrama R, Chanona G, De La Garza J (2002). Concomitant chemoradiation versus neoadjuvant chemotherapy in locally advanced cervical carcinoma: results from two consecutive phase II studies. Ann Oncol.

[24] Keys HM, Bundy BN, Stehman FB, Muderspach LI, Chafe WE, Suggs CL, Walker JL, Gersell D (1999). Cisplatin, radiation, and adjuvant hysterectomy compared with radiation and adjuvant hysterectomy for bulky stage IB cervical carcinoma. N Engl J Med.

[25] Teh J, Yap SP, Tham I, Sethi VK, Chua EJ, Yeo R, Ho TH, Tay EH, Chia YN, Soh LT, Khoo-Tan HS (2010). Concurrent chemoradiotherapy incorporating high-dose rate brachytherapy for locally advanced cervical carcinoma: survival outcomes, patterns of failure, and prognostic factors. Int J Gynecol Cancer.

[26] Pesee M, Krusun S, Padoongcharoen P (2010). High dose rate cobalt-60 afterloading intracavitary therapy for cervical carcinoma in Srinagarind hospital - analysis of survival. Asian Pac J Cancer Prev.

[27] Chaudhury K, Ghosh M, Halder A, Senapati S, Chaudhury S (2013). Is transabdominal ultrasound scanning of cervical measurement in mid-trimester pregnancy a useful alternative to transvaginal ultrasound scan?. J Turk Ger Gynecol Assoc.

[28] Berghella V, Iams JD, Newman RB, Macpherson C, Goldenberg RL, Mueller-Heubach E, Caritis SN, Dombrowski MP (2004). Frequency of uterine contractions in asymptomatic pregnant women with or without a short cervix on transvaginal ultrasound scan. Am J Obstet Gynecol.

[29] Klasa-Mazurkiewicz D, Emerich J, Milczek T (2002). The evaluation how FIGO stage in cervical cancer depends on frequency of gynaecological control. Ginekol Pol.

[30] Ackermann S, Beckmann MW (2005). Accuracy of cervical cancer staging needs improvement. Am J Obstet Gynecol.

[31] Padhani AR, Ollivier L (2001). The RECIST (Response Evaluation Criteria in Solid Tumors) criteria: implications for diagnostic radiologists. Br J Radiol.

[32] Mark Taylor S, Drover C, Maceachern R, Bullock M, Hart R, Psooy B, Trites J (2010). Is preoperative ultrasonography accurate in measuring tumor thickness and predicting the incidence of cervical metastasis in oral cancer?. Oral Oncol.

[33] Green A, Dobias SB, Walters DJ, Brasier AR (1994). Tumor necrosis factor increases the rate of lipolysis in primary cultures of adipocytes without altering levels of hormone-sensitive lipase. Endocrinology.

[34] Maru DM, Kopetz S, Boonsirikamchai P, Agarwal A, Chun YS, Wang H, Abdalla EK, Kaur H, Charnsangavej C, Vauthey JN, Loyer EM (2010). Tumor thickness at the tumor-normal interface: a novel pathologic indicator of chemotherapy response in hepatic colorectal metastases. Am J Surg Pathol.

[35] Goldman BD (2000). Melanoma and tumor thickness. Arch Dermatol.

[36] Sondergaard K (1985). Depth of invasion and tumor thickness in primary cutaneous malignant melanoma. A study of 2012 cases. Acta Pathol Microbiol Immunol Scand A.

[37] Wagenaar HC, Trimbos JB, Postema S, Anastasopoulou A, van der Geest RJ, Reiber JH, Kenter GG, Peters AA, Pattynama PM (2001). Tumor diameter and volume assessed by magnetic resonance imaging in the prediction of outcome for invasive cervical cancer. Gynecol Oncol.

[38] Okuno K, Joja I, Miyagi Y, Sakaguchi Y, Notohara K, Kudo T, Hiraki Y (2002). Cervical carcinoma with full-thickness stromal invasion: relationship between tumor size on T2-weighted images and parametrial involvement. J Comput Assist Tomogr.

[39] Armato SG, Oxnard GR, Kocherginsky M, Vogelzang NJ, Kindler HL, MacMahon H (2005). Evaluation of semiautomated measurements of mesothelioma tumor thickness on CT scans. Acad Radiol.

