# Supplementary figures and images for: Tumor invasion depth is a useful pathologic assessment for predicting outcomes in cervical squamous cell carcinoma after neoadjuvant radiotherapy
Source: Diagn Pathol. 2015 Nov 4;10:200. doi: 10.1186/s13000-015-0426-6 (PMC4632273; doi:10.1186/s13000-015-0426-6)

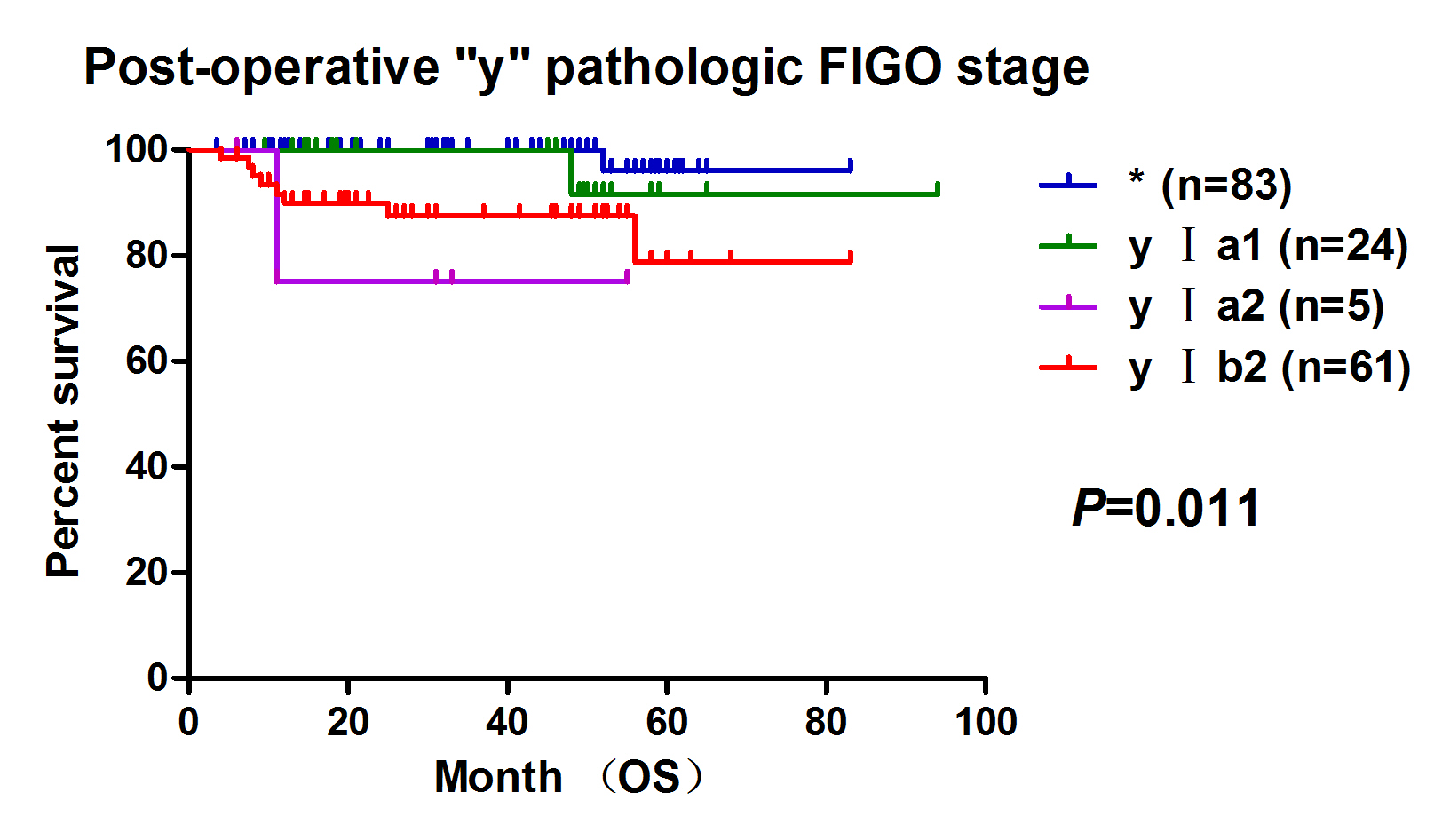

Supplement: Additional file 1: Figure S1. — Association of overall survival (OS) and progression-free survival (PFS) with the post-operative “y” Pathologic stage. (A) OS and (B) PFS curves show a slight difference with different post-operative “y” Pathologic staging after the radical hysterectomy. Note: Post-operative pathologic FIGO stage *: FIGO staging no longer includes Stage 0 (Tis). (ZIP 544 kb) [file 13000_2015_426_MOESM1_ESM.zip › Figure S1A.jpg]

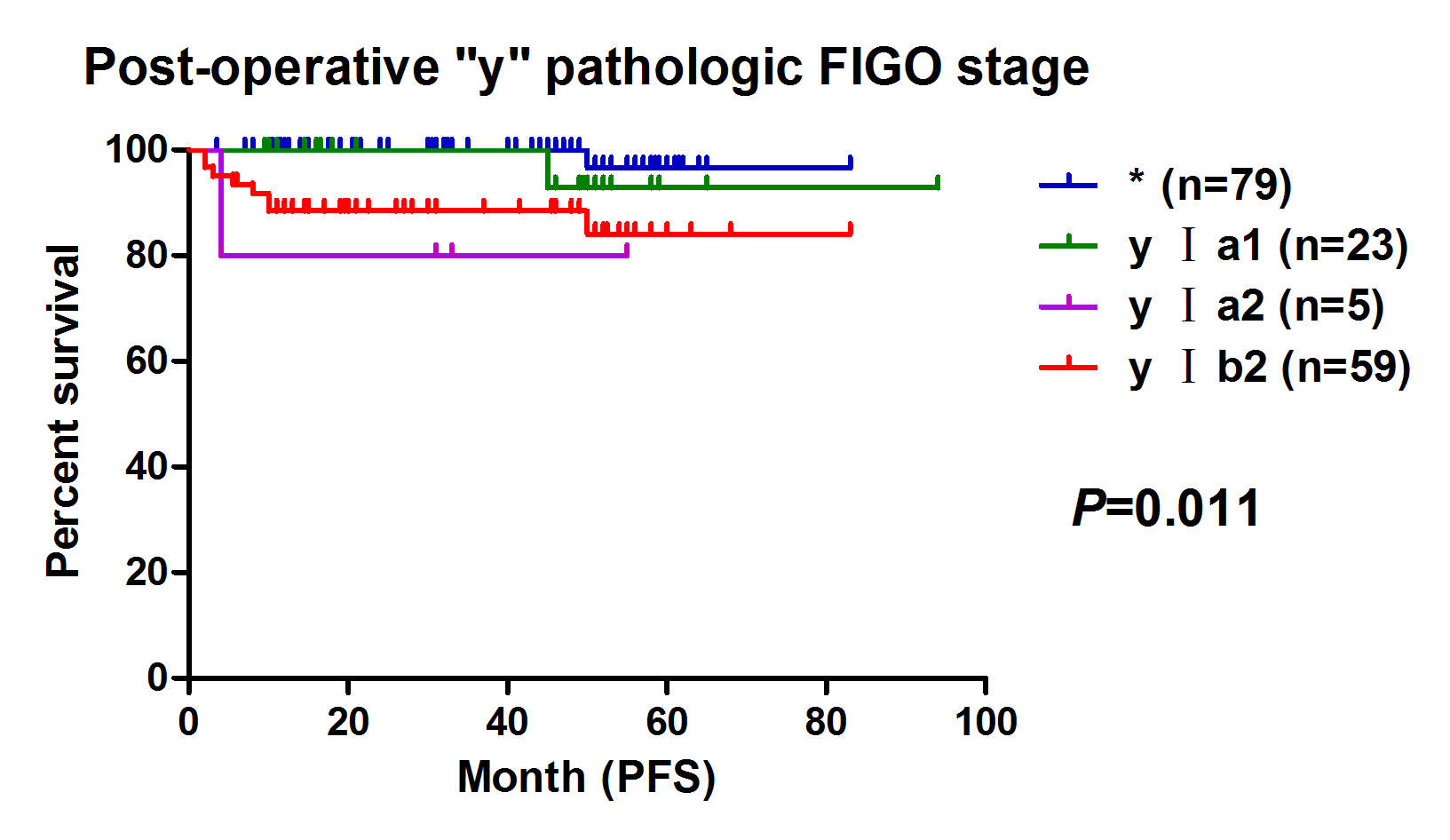

Supplement: Additional file 1: Figure S1. — Association of overall survival (OS) and progression-free survival (PFS) with the post-operative “y” Pathologic stage. (A) OS and (B) PFS curves show a slight difference with different post-operative “y” Pathologic staging after the radical hysterectomy. Note: Post-operative pathologic FIGO stage *: FIGO staging no longer includes Stage 0 (Tis). (ZIP 544 kb) [file 13000_2015_426_MOESM1_ESM.zip › Figure S1B.jpg]

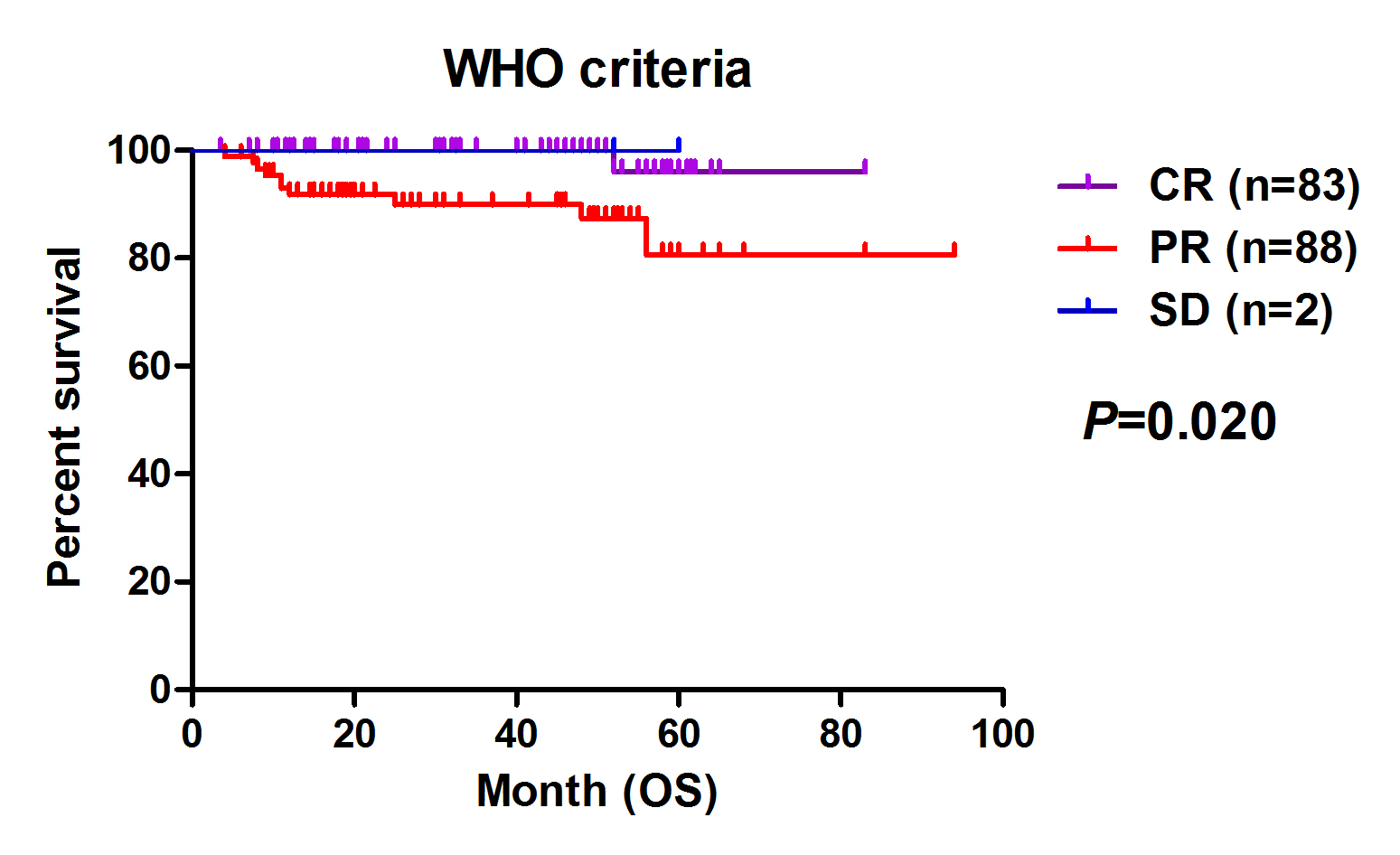

Supplement: Additional file 2: Figure S2. — Association of overall survival (OS) (A) and progression-free survival (PFS) (B) curves show a clear difference between patient outcomes according to the WHO double diameters measurement evaluation. Note: CR: Complete response, PR: Partial response, PD: Progressive disease, SD: Stable disease. (ZIP 423 kb) [file 13000_2015_426_MOESM2_ESM.zip › Figure S2A.jpg]

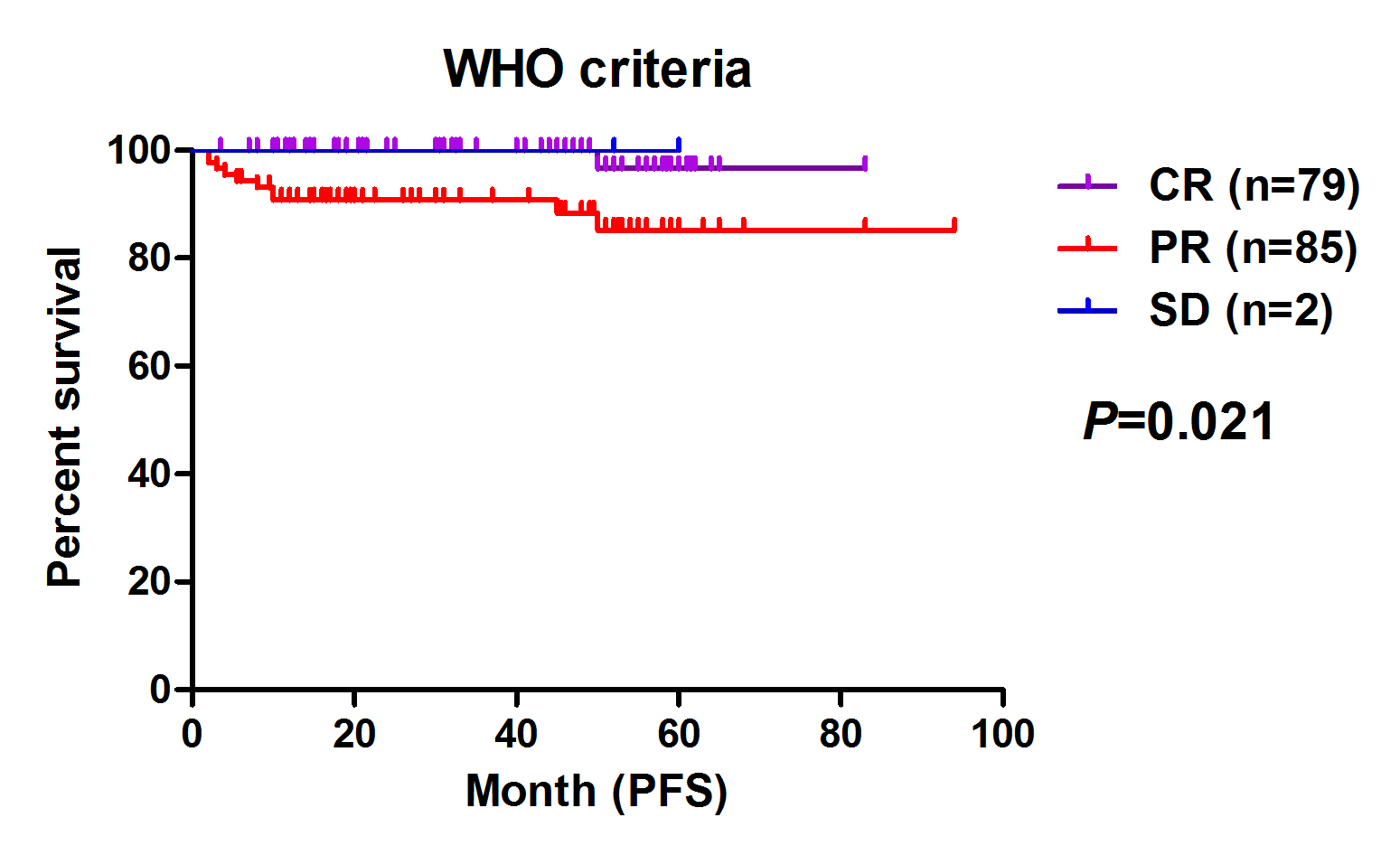

Supplement: Additional file 2: Figure S2. — Association of overall survival (OS) (A) and progression-free survival (PFS) (B) curves show a clear difference between patient outcomes according to the WHO double diameters measurement evaluation. Note: CR: Complete response, PR: Partial response, PD: Progressive disease, SD: Stable disease. (ZIP 423 kb) [file 13000_2015_426_MOESM2_ESM.zip › Figure S2B.jpg]

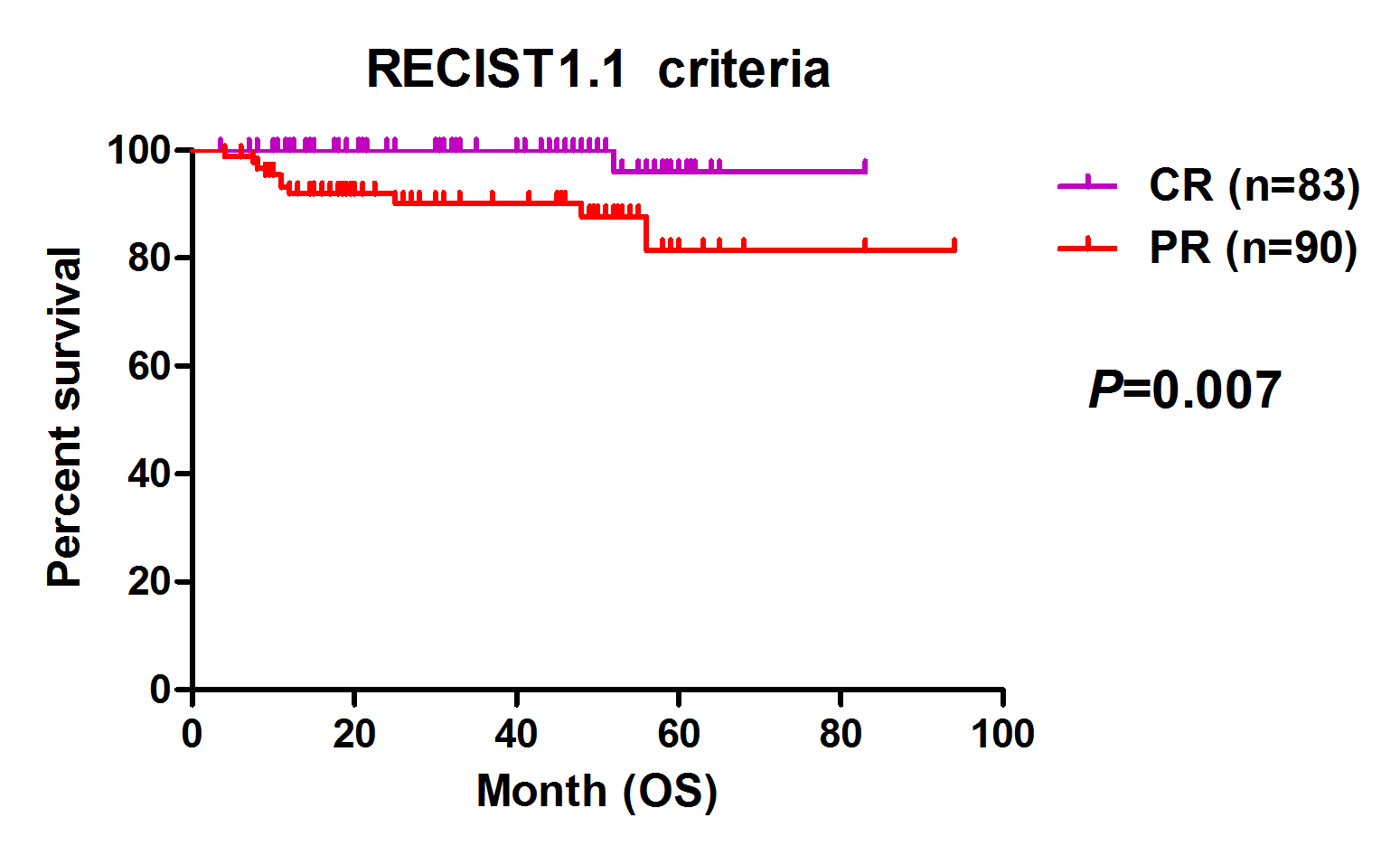

Supplement: Additional file 3: Figure S3. — Association of overall survival (OS) (A) and progression-free survival (PFS) (B) curves show a clear difference between patient outcomes according to response evaluation criteria in solid tumors (RECIST1.1). Note: CR: Complete response, PR: Partial response, PD: Progressive disease, SD: Stable disease. (ZIP 399 kb) [file 13000_2015_426_MOESM3_ESM.zip › Figure S3A.jpg]

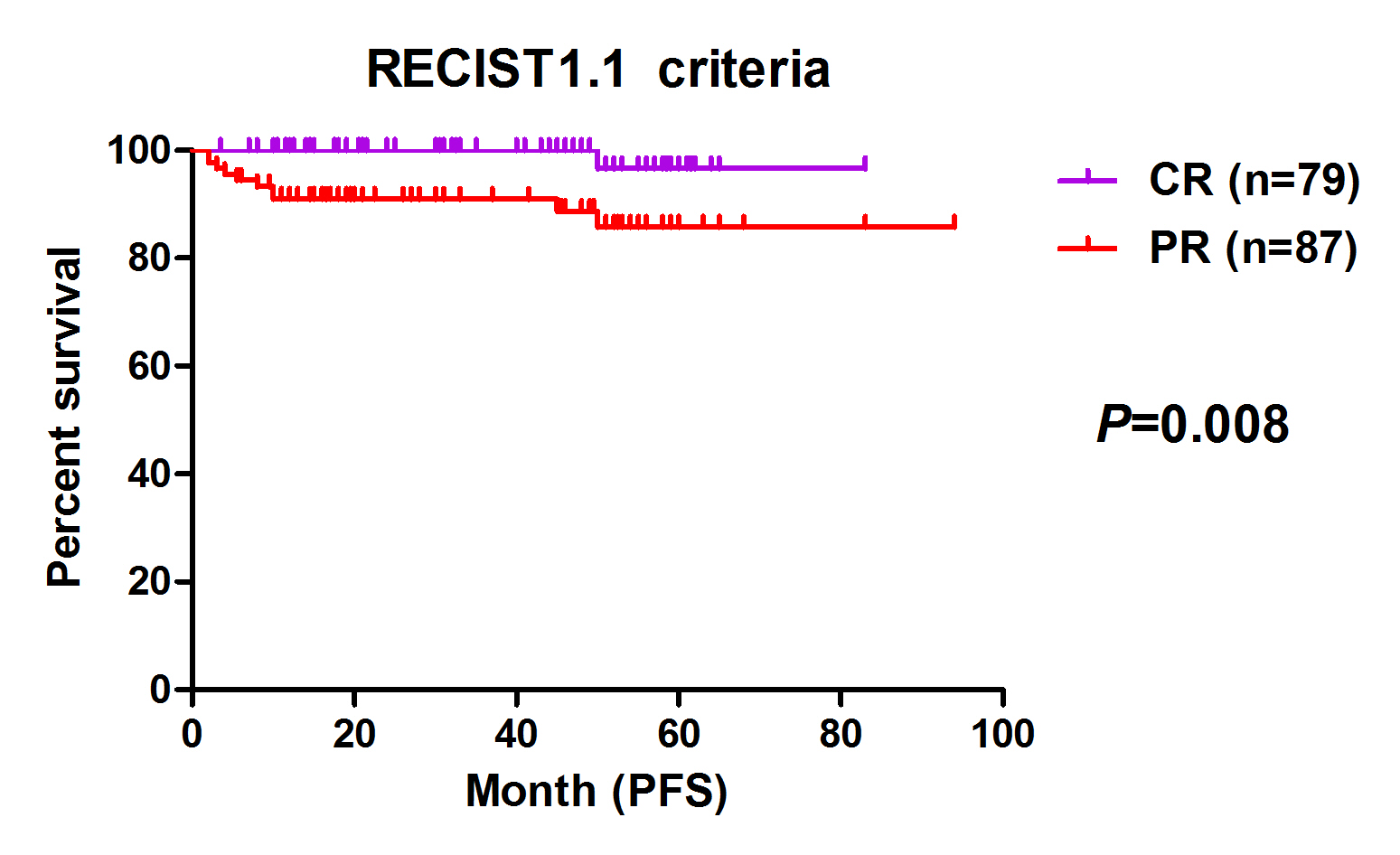

Supplement: Additional file 3: Figure S3. — Association of overall survival (OS) (A) and progression-free survival (PFS) (B) curves show a clear difference between patient outcomes according to response evaluation criteria in solid tumors (RECIST1.1). Note: CR: Complete response, PR: Partial response, PD: Progressive disease, SD: Stable disease. (ZIP 399 kb) [file 13000_2015_426_MOESM3_ESM.zip › Figure S3B.jpg]
